# A Comparative Study of Functional Outcomes Between Conservative Management and Volar Locking Plate Fixation in Extra-articular Distal Radius Fractures

**DOI:** 10.7759/cureus.96496

**Published:** 2025-11-10

**Authors:** Arjun Singh Sastya, Abhilekh Mishra, Yogesh Singh Parihar

**Affiliations:** 1 Department of Orthopaedics and Traumatology, Gajra Raja Medical College, Gwalior, IND

**Keywords:** conservative treatment, distal radius fracture, extra-articular fracture, functional outcome, volar locking plate

## Abstract

Background

Fractures of the distal radius are among the most common upper limb injuries. Management options range from closed reduction with casting to open reduction and internal fixation (ORIF) using volar locking plates. The optimal treatment approach for extra-articular fractures remains debated.

Objective

The objective of this study was to compare functional and radiological outcomes between conservative treatment and volar locking plate fixation in extra-articular distal radius fractures.

Methods

A prospective comparative cohort study was conducted on 60 patients with extra-articular distal radius fractures, allocated into two equal groups: Group A underwent closed reduction with plaster immobilization, and Group B underwent ORIF with a volar locking plate. Functional outcomes were assessed using the Green and O’Brien scoring system at regular intervals up to six months. Radiological evaluation included radial length, volar tilt, and radial inclination. Complications were recorded. Statistical analysis was performed using the chi-square test and independent t-test, with p<0.05 considered significant.

Results

The mean age was comparable between groups (p>0.05). At final follow-up, Group B demonstrated significantly better mean Green and O’Brien scores (88.2 ± 6.1) compared to Group A (76.5 ± 8.4) (p<0.001). Radiological parameters were closer to anatomical norms in Group B (p<0.05 for radial length and volar tilt). Complications were more frequent in Group A, including malunion and stiffness, whereas Group B had occasional implant-related irritation.

Conclusion

Within the limits of a non-randomized design, volar locking plate fixation yields superior functional and radiological outcomes compared to conservative treatment for displaced extra-articular distal radius fractures, with fewer late complications and earlier return of function.

## Introduction

Fractures of the distal radius remain a frequent and clinically important orthopaedic problem; modern management strategies, including fixed-angle volar locking plate systems, have been developed to improve anatomical restoration and functional recovery [1]. Contemporary comparative trials and prospective series have examined different fixation strategies, including fixed-angle volar plates, external fixation, and percutaneous pinning, to identify approaches that minimize loss of reduction while maximizing early function [2,3]. Distal radius fractures occur across the lifespan, often following low-energy falls in older adults and high-energy trauma in younger individuals, and they contribute substantially to disability and healthcare utilization worldwide [3,4]. Colles first described the distal radius injury pattern in the 19th century [1[, and the subsequent evolution of treatment has focused on balancing ease of care with maintenance of anatomy and function [4,5].

Conservative treatment with closed reduction and plaster immobilization remains an important option for minimally displaced and stable fracture patterns because of its accessibility and low immediate cost. However, casting may be associated with late collapse, malunion, and functional deficits in fractures that are predisposed to instability [5]. Classical prognostic factors for instability, such as dorsal comminution, dorsal angulation, loss of radial height, and patient age/osteoporosis, have been described and are commonly used to guide treatment selection [6,7]. These concerns have led to wider adoption of surgical fixation methods intended to maintain reduction and permit early mobilization.

Among operative strategies, volar locking plate fixation has become increasingly popular for unstable extra-articular and selected intra-articular fractures because it provides angular stability, facilitates anatomic realignment, and supports early rehabilitation [8-10]. Biomechanical comparisons have demonstrated that modern volar locking constructs resist axial and torsional loads more effectively than many older dorsal or non-locking constructs [11]. Clinical series and randomized studies have reported improved early outcomes and reduced redisplacement rates with volar plating when compared with external fixation or percutaneous techniques [9,10,12]. Pragmatic considerations regarding implant selection (for example, plate profile and diameter) have also been evaluated in comparative series, informing the choice between 2.4-mm and larger systems.

In the Indian and similar low- and middle-income country (LMIC) healthcare settings, socioeconomic constraints, delayed presentation, and restricted access to specialized surgical services often influence the decision between conservative and surgical treatment [13]. Published local series comparing percutaneous fixation and casting, as well as procedure-specific reports, highlight the need for context-specific outcome data to guide resource-appropriate management [13,14]. Long-term follow-up studies have shown that residual deformity and malunion following conservative treatment can lead to persistent pain, stiffness, and functional limitation, underscoring the importance of achieving anatomical alignment even in low-resource settings [15]. Furthermore, comparative analyses of volar plate systems have explored design characteristics, such as plate thickness and screw configuration, providing evidence that low-profile, fixed-angle plates yield improved outcomes and fewer hardware-related complications [16]. Randomized and prospective investigations from other settings provide useful benchmarks, but direct comparative data within local practice environments remain limited [17,18].

The Green and O’Brien scoring system, modified by Cooney et al. [19], remains one of the most widely used functional assessment tools for evaluating clinical outcomes following distal radius fracture management. Comparative studies and multicenter trials have demonstrated that volar locking plate fixation allows superior anatomical reduction and early mobilization compared to percutaneous or external fixation methods [20,21]. These findings highlight the functional advantages of stable internal fixation in achieving earlier wrist motion and faster recovery.

Subsequent high-level evidence has further consolidated these observations. Recent systematic reviews and meta-analyses have continued to support these findings, demonstrating that volar locking plate fixation provides more reliable maintenance of reduction, faster functional recovery, and earlier return to daily activity compared with conservative management [22-24]. These contemporary studies reinforce the ongoing relevance of evaluating both treatment strategies within diverse clinical contexts, particularly in resource-constrained healthcare systems such as India.

The present report is a prospective comparative cohort study designed to evaluate the functional and radiological outcomes of conservative management versus volar locking plate fixation in extra-articular distal radius fractures. The primary objective was to compare functional recovery using the Green and O’Brien score (modified by Cooney), and the secondary objective was to compare radiological restoration (radial length, volar tilt, and radial inclination) on standardized posteroanterior and lateral radiographs. We hypothesized that volar locking plate fixation would achieve more rapid functional recovery and better maintenance of anatomical alignment than conservative plaster immobilization.

## Materials and methods

Study design and setting

This was a prospective, non-randomized comparative clinical study conducted in the Department of Orthopaedics, Gajra Raja Medical College, Gwalior, India, from May 2020 to November 2021. The study protocol was approved by the Institutional Ethics Committee, Gajra Raja Medical College (approval number: 76/IEC-GMRC/2019). All participants provided written informed consent before enrollment.

Eligibility criteria

Adults (≥18 years) presenting within seven days of injury with displaced extra-articular distal radius fractures (AO type A2/A3) were eligible. Exclusion criteria were intra-articular fractures (AO type B/C), open or pathological fractures, prior deformity or fracture of the ipsilateral wrist, and associated ipsilateral upper-limb fractures.

Enrollment and allocation

Eligible patients were consecutively enrolled and allocated into two treatment groups: Group A (Conservative Management) and Group B (Volar Locking Plate Fixation). As this was a non-randomized comparative study, allocation was determined by a combination of patient preference and surgeon judgment. Patient preference was obtained following structured counseling, where both treatment options were explained in terms of expected recovery, potential complications, costs, and rehabilitation timelines. Preferences were recorded in a standardized consent form signed by the patient and the investigator. Surgeon judgment was guided by predefined clinical and radiographic criteria, including fracture stability (as per Lafontaine criteria [6]), bone quality, soft-tissue condition, comorbidities affecting anesthesia or healing potential, and occupational or functional demand.

To minimize selection bias, baseline demographic and injury characteristics (age, sex, side, and trauma mechanism) were compared between groups and found to be statistically similar (p > 0.05).

Sample size calculation

The sample size was determined based on an anticipated mean difference of 10 points in the Cooney modification of the Green and O’Brien functional score between groups, with a standard deviation of 10 points derived from prior comparative studies [2,9,12]. Using a power of 80% and a two-sided α = 0.05, the minimum sample size required was 25 patients per group. To account for possible attrition, 60 patients (30 per group) were included. Sixty consecutive eligible patients were enrolled and allocated into two equal groups of 30 each: Group A received closed reduction and below-elbow cast immobilization; Group B underwent open reduction and internal fixation (ORIF) with a volar locking plate. 

Interventions

Group A

Conservative management: Closed reduction was performed under a hematoma block or regional anesthesia using the three-point pressure principle and the modified Kapandji technique to correct dorsal angulation and restore radial height under fluoroscopic guidance. A below-elbow plaster of Paris cast was applied in neutral to 10° flexion and 15° ulnar deviation to maintain physiological palmar tilt. The reduction was considered acceptable when: Radial height loss ≤ 5 mm, Radial inclination ≥ 15°, and Volar tilt was between 0° and 15°.

Radiographs were obtained immediately after reduction and at each follow-up (one, two, and four weeks) to confirm stability. Re-manipulation or conversion to surgical fixation was indicated if there was >10° loss of volar tilt or >5 mm loss of radial height during follow-up. Immobilization was maintained for six weeks, followed by structured physiotherapy emphasizing a progressive range of motion (ROM) and grip strengthening.

Group B

Volar locking plate fixation: ORIF was performed through a standard volar approach centered on the flexor carpi radialis (FCR) interval. Reduction was achieved under fluoroscopic guidance, and fixation was performed using a pre-contoured 2.4-mm volar locking plate with distal locking and proximal cortical screws. Tourniquet control, careful soft-tissue handling, and appropriate screw length selection were emphasized to prevent flexor tendon irritation [8-10]. Outcome assessors were not blinded to treatment allocation. All patients received prophylactic antibiotics as per institutional policy. Postoperatively, the wrist was supported in a removable splint for one to two weeks for comfort.

Perioperative and rehabilitation protocols

A standardized, structured rehabilitation regimen was applied to both groups. All patients were followed by the same physiotherapy team to ensure uniformity, and compliance was reinforced during each review.

Conservative Group

Active wrist and forearm mobilization exercises began after cast removal (at six weeks), progressing to resistive and strengthening exercises under physiotherapist supervision.

Surgical Group

Active finger and elbow motion was encouraged from postoperative day 1. Gentle wrist mobilization began after suture removal (10-14 days), progressing to full active ROM by 10-12 weeks.

Outcomes and measurements

Primary Outcome

Functional recovery was assessed using the Green and O’Brien score (modified by Cooney) [19], which includes four domains: pain, function, ROM, and grip strength. Scores were recorded at six weeks, three months, and six months post intervention.

Secondary Outcomes

Radiological parameters, including radial length (ulnar variance), radial inclination, and volar tilt, were measured on standardized posteroanterior and lateral radiographs following accepted definitions in the distal radius literature [3-5].

Complications such as malunion, stiffness, infection, and implant irritation were prospectively recorded throughout follow-up. Follow-up schedule: Clinical and radiographic assessments were performed at two weeks, six weeks, three months, and six months.

Statistical analysis

Statistical analysis was performed using IBM SPSS Statistics for Windows, Version 22.0 (IBM Corp., Armonk, New York, United States) and cross-verified using R version 4.3 (R Foundation for Statistical Computing, Vienna, Austria, https://www.R-project.org/). Continuous variables were expressed as mean ± standard deviation (SD), and categorical variables as frequencies and percentages.

Given the non-randomized, repeated-measures design, linear mixed-effects models (LMM) were used to analyze outcomes measured over time (ROM, grip strength, Green and O’Brien scores). Each model included fixed effects for group, time, and their interaction (group × time), and random intercepts for subjects to account for within-patient correlation and missing data.

For single-time-point comparisons, analysis of covariance (ANCOVA) was applied, adjusting for baseline covariates (age, sex, side of injury, and trauma mechanism). Binary outcomes (complications) were analyzed using log-binomial regression with robust variance estimation to obtain adjusted risk ratios.

Sensitivity analyses assessed model assumptions, covariate balance, and robustness to variable inclusion. Statistical significance was defined as p < 0.05 for all tests.

Control of confounders

Demographic and injury-related variables (age, sex, side, and mechanism of trauma) were compared between groups at baseline to confirm comparability. Adjusted models were used to control for these covariates in both continuous and categorical outcome analyses, mitigating confounding effects related to the non-randomized design.

## Results

Demographics 

Sixty patients were enrolled, with 30 in each group. The mean age was similar between groups (41.2 ± 12.4 years in Group A vs. 39.8 ± 11.9 years in Group B, p=0.65), and the majority were male (60% in Group A, 66.7% in Group B). Right-sided injuries predominated in both groups, and high-energy trauma (e.g., road traffic accidents) accounted for approximately 40% of cases (Table 1). These demographic patterns are consistent with the epidemiological profile reported by Chung and Spilson [2], with a higher proportion of high-energy injuries in the younger male subset.

**Table 1:** Demographic characteristics of patients

Parameter	Group A (Conservative), n=30	Group B (Volar Plating), n=30
Mean age (years)	41.2 ± 12.4	39.8 ± 11.9
Male	18 (60%)	20 (66.7%)
Right side involved	17 (56.7%)	18 (60%)
High-energy trauma	12 (40%)	13 (43.3%)

Range of motion

**Figure 1:**
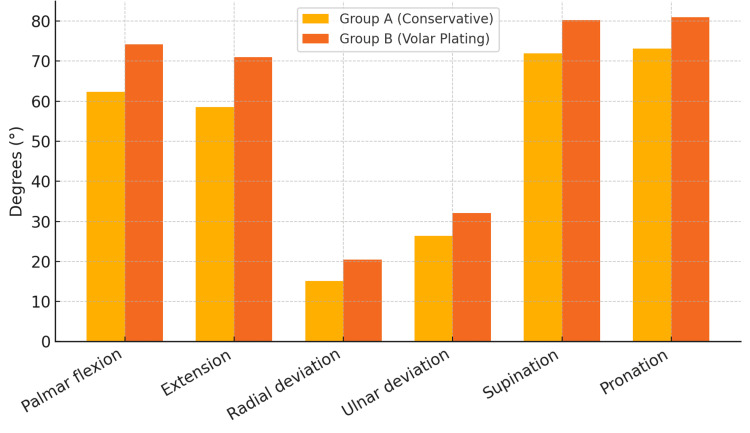
Range of motion comparison at six months

At six months, Group B (volar plating) showed greater ROM in all planes (all p < 0.001) (Table 2, Figure 1). The largest gains were in palmar flexion/extension, consistent with early functional recovery after plating reported by Wilcke et al. [11]. Restoration of radial length and volar tilt, key determinants of wrist biomechanics, has been emphasized by Lafontaine et al. [4] and Abbaszadegan et al. [5].

**Table 2:** Range of motion at six months Independent t-test was applied to compare mean degrees of movement (palmar flexion, extension, radial deviation, ulnar deviation, supination, pronation) between the groups. Corresponding t-values and p-values are shown.

Movement	Group A, mean ± SD	Group B, mean ± SD	t-value	p-value
Palmar flexion (°)	62.3 ± 6.8	74.2 ± 5.6	7.40	<0.001
Extension (°)	58.5 ± 7.2	71.0 ± 6.0	7.31	<0.001
Radial deviation (°)	15.1 ± 2.8	20.4 ± 3.0	7.07	<0.001
Ulnar deviation (°)	26.4 ± 3.4	32.1 ± 3.6	6.30	<0.001
Supination (°)	72.0 ± 5.9	80.3 ± 4.8	5.98	<0.001
Pronation (°)	73.2 ± 6.2	81.0 ± 4.4	5.62	<0.001

Grip strength

**Figure 2:**
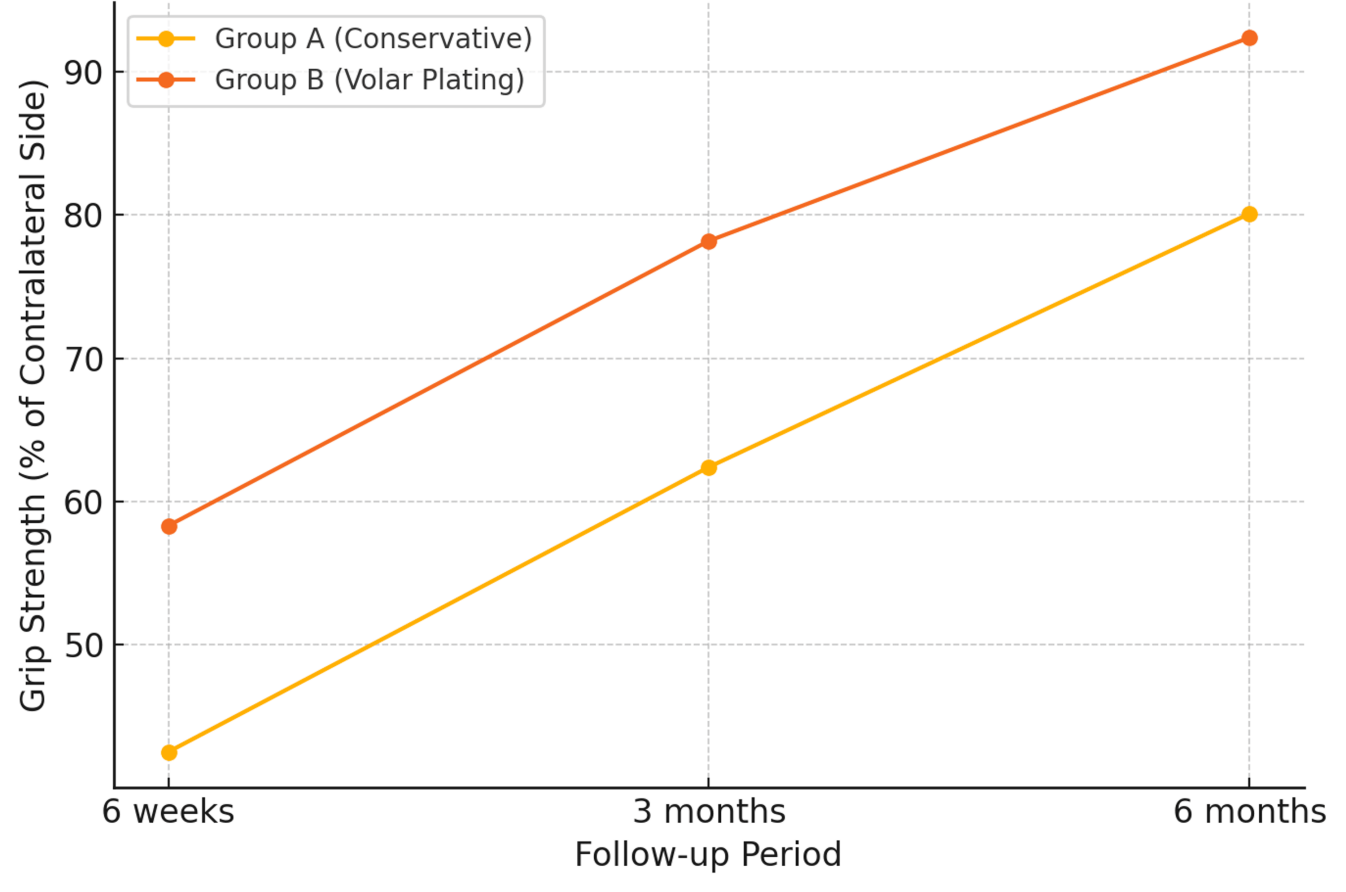
Trend showing grip strength recovery over time

Grip strength recovery was faster and more complete in the plating group. By six months (Figure 2), Group B achieved 92.4% of contralateral grip strength compared to 80.1% in Group A (p<0.001) (Table 3). Early mobilization after stable fixation likely contributed to this difference, similar to the accelerated functional recovery noted in Rizzo et al. [8] and Nimmagadda et al. [12].

**Table 3:** Grip strength as percentage of contralateral side Independent t-test was used to compare the mean grip strength at six weeks, three months, and six months between groups. Corresponding t-values and p-values are shown.

Time Point	Group A (%), mean ± SD	Group B (%), mean ± SD	t-value	p-value
6 weeks	42.5 ± 6.1	58.3 ± 5.8	10.28	<0.001
3 months	62.4 ± 5.4	78.2 ± 5.6	11.12	<0.001
6 months	80.1 ± 4.7	92.4 ± 3.2	11.85	<0.001

Functional scores

**Figure 3:**
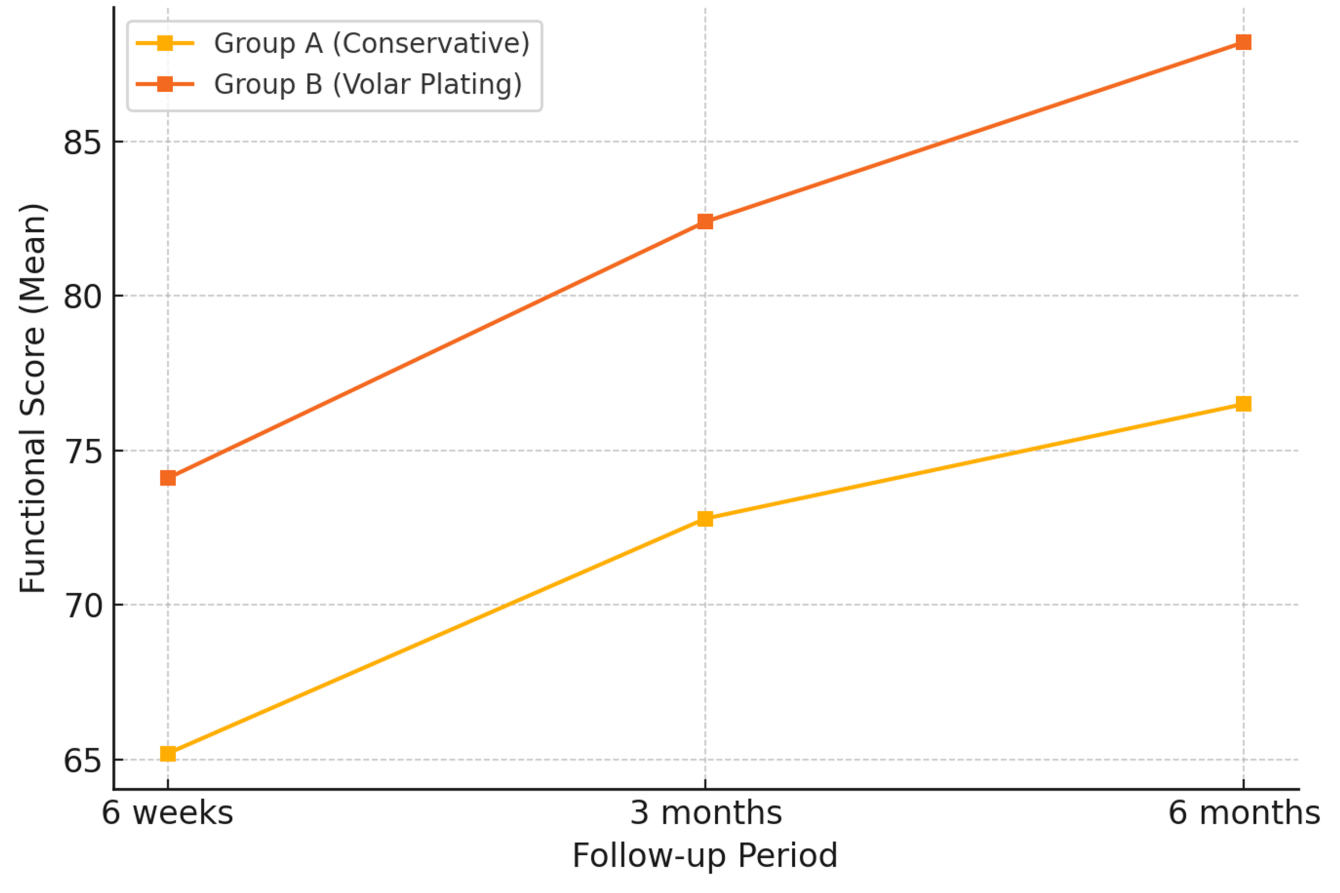
Green & O’Brien functional scores over time

Using the Green and O’Brien scoring system modified by Cooney et al. (Figure 3), Group B had significantly higher scores in pain relief, functional use, range of motion, and grip strength domains (p<0.001 for all parameters) (Table 4). The mean total score in the surgical group (88.2 ± 6.1) indicated a predominance of excellent-to-good outcomes, aligning with results from Orbay and Fernandez [7] and Figl et al. [9], who both reported improved early function with volar plating.

**Table 4:** Functional scores (Green and O’Brien, modified by Cooney) Independent t-test was used for each subdomain (pain, function, range of motion, grip strength) and total scores.

Parameter	Group A, mean ± SD	Group B, mean ± SD	t-value	p-value
Pain	20.1 ± 2.0	24.0 ± 1.4	8.74	<0.001
Functional use	19.0 ± 1.8	23.5 ± 1.2	11.36	<0.001
Range of motion	18.4 ± 1.6	21.5 ± 1.3	8.18	<0.001
Grip strength	19.0 ± 1.7	22.2 ± 1.5	7.67	<0.001
Total score	76.5 ± 8.4	88.2 ± 6.1	5.96	<0.001

Radiological outcomes

**Figure 4:**
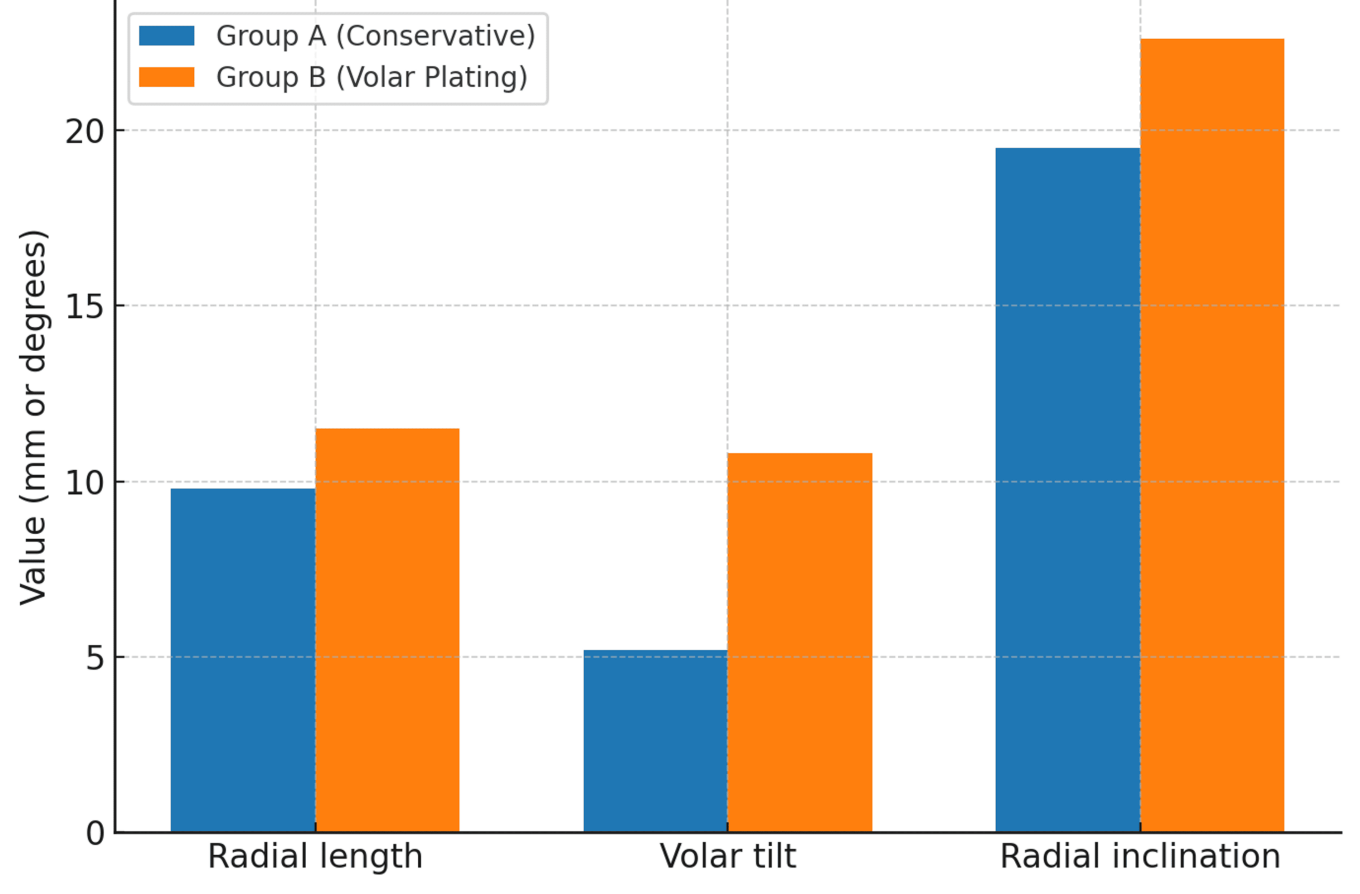
Radiological parameters at final follow-up

Radiographic evaluation at six months (Figure 4) showed that Group B had values closer to normal anatomical parameters: radial length (11.5 mm vs. 9.8 mm), volar tilt (10.8° vs. 5.2°), and radial inclination (22.6° vs. 19.5°) (p<0.001 for all) (Table 5). Maintenance of these parameters reduces the risk of altered wrist mechanics and subsequent degenerative changes, as previously described by Lafontaine et al. [4] and Abbaszadegan et al. [5].

**Table 5:** Radiological parameters at final follow-up Independent t-test was used to compare mean radial length, volar tilt, and radial inclination.

Parameter	Group A, mean ± SD	Group B, mean ± SD	t-value	p-value
Radial length (mm)	9.8 ± 1.2	11.5 ± 0.9	6.30	<0.001
Volar tilt (°)	5.2 ± 3.0	10.8 ± 2.5	7.84	<0.001
Radial inclination (°)	19.5 ± 2.3	22.6 ± 1.9	5.71	<0.001

Complications

Complication rates were low in both groups, but differed in nature. Malunion occurred in 13.3% of conservatively treated patients, consistent with reports from Villar et al. [14], who emphasized the influence of casting position on final alignment and risk of stiffness. No malunions occurred in the plating group. Implant-related irritation occurred in two surgically treated patients but was managed with hardware removal. Superficial infections were rare and equally distributed (Table 6). These findings mirror the complication profiles in Rizzo et al. [8] and Rozental et al. [18], where surgical fixation reduced malunion but introduced hardware-related issues.

**Table 6:** Complications

Complication	Group A (n=30), n (%)	Group B (n=30), n (%)
Malunion	4 (13.3%)	0 (0%)
Stiffness	5 (16.7%)	1 (3.3%)
Implant-related irritation	0 (0%)	2 (6.7%)
Superficial infection	1 (3.3%)	1 (3.3%)

## Discussion

This prospective comparative cohort study evaluated the functional and radiological outcomes of volar locking plate fixation versus conservative plaster immobilization for displaced extra-articular distal radius fractures. The findings demonstrate that volar plate fixation was associated with significantly better range of motion, grip strength, and Green & O’Brien functional scores, as well as superior restoration of anatomical parameters, when compared to conservative treatment. These observations are consistent with previous randomized and observational studies highlighting the biomechanical stability and early rehabilitation benefits of volar plating [8-10,12,20].

Comparison with previous studies

Our results are in agreement with McFadyen et al. [20], who found superior early functional outcomes with volar plating compared to Kirschner-wire fixation. Similarly, Rizzo et al. [8] and Figl et al. [9] reported improved radiological alignment and lower complication rates with volar locking plates relative to external fixation. The multicenter VIPER trial by Mulders et al. further substantiated these findings, demonstrating faster recovery and better patient-reported outcomes in surgically managed fractures [21]. More recent meta-analyses have reinforced this evidence: Yang et al. [22] and Zhu et al. [23] confirmed that volar plating provides superior early functional recovery and anatomical restoration compared with casting, while a 2022 systematic review in European Orthopaedics and Traumatology Review [24] emphasized reduced redisplacement and earlier return to activity. Collectively, these studies strengthen the external validity of our findings and confirm that volar plate fixation continues to outperform conservative management, particularly in unstable fracture configurations.

Socioeconomic and accessibility factors often influence treatment choice. The accelerated recovery observed in our study mirrors the outcomes reported by Nimmagadda et al. [12], while older studies by Gupta [13] and Villar et al. [14] demonstrated higher malunion and stiffness rates with prolonged casting. Given that many patients in our population rely on early return to manual labor, the early mobilization enabled by volar plating has significant functional implications. However, we also acknowledge that surgical costs and implant availability remain limiting factors in many public healthcare institutions.

Complication profile

Complications were infrequent in both groups but differed in type. Malunion occurred exclusively in the conservative group (13.3%), consistent with Villar et al. [14], while minor hardware irritation was observed in two surgical cases [8,18], managed successfully with elective implant removal. These findings parallel those of Rozental et al. [18] and Rizzo et al. [8], illustrating that plating reduces deformity-related complications at the cost of occasional hardware issues. From an economic standpoint, recent analyses show mixed results: Mulders et al. [21] reported that volar plate fixation was cost-effective due to faster return to work and improved short-term utility, whereas Saving et al. [25] found that although volar plating offered superior early function, it incurred marginally higher long-term costs with minimal quality-adjusted life year (QALY) gains compared to external fixation. This highlights the importance of individualized decision-making based on patient demands and local resource availability.

Interpretation and clinical relevance

The present findings strengthen the evidence that anatomical reduction and stable fixation are critical determinants of optimal wrist function following distal radius fractures. Although both techniques remain viable, conservative management is best reserved for stable or minimally displaced patterns, whereas surgical fixation appears preferable for displaced or potentially unstable fractures, particularly in patients requiring rapid return to activity.

However, these findings should be interpreted with appropriate caution. The study’s non-randomized design introduces potential selection bias, as patients opting for surgery may differ in age, bone density, activity level, or motivation compared to those managed conservatively. Thus, while our results demonstrate a strong association between volar plate fixation and improved outcomes, causation cannot be definitively established.

The consistency of our results with higher-level evidence, such as randomized trials by Grewal et al. [17], Marcheix et al. [18], and the VIPER trial [21], supports the external validity of our findings and suggests that the observed benefits are both biologically and clinically plausible. Nevertheless, definitive conclusions regarding therapeutic superiority require confirmation through larger, randomized controlled studies.

This study’s strengths include its prospective design, standardized follow-up, and the use of validated functional (Green and O’Brien) and radiological outcome measures, ensuring reproducibility and reliability. Both conservative and surgical protocols were clearly defined and supervised by experienced surgeons, minimizing procedural variability.

Limitations

Despite its strengths, this study has several important limitations that must be acknowledged. First, the non-randomized design and allocation based on patient preference and surgeon judgment introduce a degree of selection bias, as patients choosing surgery may differ systematically from those managed conservatively in factors such as age, activity level, or socioeconomic background. Although baseline demographic and injury variables were statistically comparable and statistical adjustments were applied, residual confounding cannot be excluded. This limitation affects the internal validity and restricts the ability to infer causality.

Second, blinding of outcome assessors was not performed. Functional evaluations using the Green and O’Brien score and radiographic measurements were conducted by investigators aware of treatment assignment, which may have introduced observer bias, particularly for subjective outcome domains such as pain and functional use. Future studies should incorporate blinded or independent assessors to minimize this risk.

Third, this was a single-center study conducted at a tertiary care institution in Gwalior, India. While this provides valuable insight into treatment outcomes within an Indian clinical context, it limits external validity and the generalizability of results to other healthcare systems with differing patient demographics, rehabilitation access, or surgical protocols.

Fourth, although the study achieved complete follow-up for all 60 participants, the follow-up duration of six months represents early to mid-term recovery. Longer-term outcomes, particularly regarding post-traumatic arthritis, implant-related issues, or late functional decline, could not be evaluated. A two-year or longer follow-up would better determine the persistence of functional and radiological advantages observed with volar plating.

Finally, while validated outcome measures and appropriate statistical modeling were employed, residual unmeasured confounding remains a limitation inherent to observational designs. Despite these constraints, the study provides meaningful comparative data and serves as a basis for larger, multicenter randomized trials to further define optimal treatment strategies for extra-articular distal radius fractures.

## Conclusions

Within the limitations of a non-randomized cohort design, this study found that volar locking plate fixation was associated with better early functional and radiological outcomes than conservative plaster immobilization for displaced extra-articular distal radius fractures. These findings align with contemporary meta-analyses and international guidelines, reinforcing that stable internal fixation facilitates early mobilization and anatomical restoration. However, the absence of randomization and blinding precludes definitive causal inference. The results should therefore be interpreted as complementary observational evidence that supports, rather than replaces, high-level randomized data-particularly relevant for clinical decision-making in resource-limited healthcare environments.
